# Effect of the transdermal low-level laser therapy on endothelial function

**DOI:** 10.1007/s10103-016-1971-2

**Published:** 2016-06-14

**Authors:** Alicja Szymczyszyn, Adrian Doroszko, Ewa Szahidewicz-Krupska, Piotr Rola, Radosław Gutherc, Jakub Jasiczek, Grzegorz Mazur, Arkadiusz Derkacz

**Affiliations:** 1 Department of Internal and Occupational Diseases, Hypertension and Clinical Oncology, Wroclaw Medical University, Borowska 213 Street, 50-552 Wroclaw, Poland; 2 Research and Development Department, Wrovasc – Integrated Cardiovascular Centre Provincial Specialist Hospital in Wroclaw, Kamienskiego 73a Street, 51-124 Wroclaw, Poland

**Keywords:** Low-level laser therapy (LLLT), Vascular endothelium, Nitric oxide metabolism, Oxidative stress

## Abstract

The effect of low-level laser therapy (LLLT) on the cardiovascular system is not fully established. Since the endothelium is an important endocrine element, establishing the mechanisms of LLLT action is an important issue.The aim of the study was to evaluate the effect of transdermal LLLT on endothelial function.In this study, healthy volunteers (*n* = 40, age = 20–40 years) were enrolled. *N* = 30 (14 female, 16 male, mean age 30 ± 5 years) constituted the laser-irradiated group (LG). The remaining 10 subjects (6 women, 4 men, mean age 28 ± 5 years) constituted the control group (CG). Participants were subjected to LLLT once a day for three consecutive days. Blood for biochemical assessments was drawn before the first irradiation and 24 h after the last session. In the LG, transdermal illumination of radial artery was conducted (a semiconductor laser λ = 808 nm, irradiation 50 mW, energy density 1.6 W/cm^2^ and a dose 20 J/day, a total dose of 60 J). Biochemical parameters (reflecting angiogenesis: vascular endothelial growth factor (VEGF), fibroblast growth factor (FGF), angiostatin; antioxidative status: glutathione (GSH) and the nitric oxide metabolic pathway: symmetric dimethylarginine (SDMA), asymmetric dimethylarginine (ADMA) and l-arginine) were assessed. In the LG, a significant increase in GSH levels and considerable decrease in angiostatin concentration following the LLLT were observed. No significant differences in levels of the VEGF, FGF, SDMA, ADMA were observed.LLLT modifies vascular endothelial function by increasing its antioxidant and angiogenic potential. We found no significant differences in levels of the nitric oxide pathway metabolites within 24 h following the LLLT irradiation.

## Introduction

The effect of low-level laser therapy (LLLT) on blood vessels and streaming blood is not fully established. Endothelial cells play a pivotal role in the maintenance of cardiovascular homeostasis due to the paracrine regulation of haemostasis, inflammation and vascular tone. Endothelial dysfunction is a well-established factor for the development of cardiovascular disease and, nowadays, is considered to be the first pathogenic link leading to its development [1]. One of the crucial factors affecting the normal endothelial function is its redox status. An antioxidative imbalance resulting from increased oxidative stress induces abnormal regulation of the haemostatic processes, promotes vasoconstriction by releasing the cytokines activating vascular smooth muscles, induces inflammatory reaction and changes vascular proliferative potential [2]. Increased oxidative stress results in scavenging nitric oxide, a potent vasodilatory and anti-inflammatory compound, leading to formation of peroxynitrites, which in turn promote S-nitration and nitrosylation of amino acid residues leading to changes in protein function and stability. Increased proteolysis is a source of methylated amino acids, i.e. asymmetrical dimethylarginine (ADMA), which is a potent inhibitor of nitric oxide synthase (NOS) and is considered to be an independent cardiovascular risk factor. Symmetrical dimethylarginine does not exert inhibitory effect on the NOS and is a marker of early kidney damage by oxidative stress. All the abnormalities mentioned above change the nitric oxide bioavailability by shifting the balance between its synthesis and degradation, and it may in turn promote inflammation and modify the proliferative/antiproliferative balance of the vascular bed [3]. The proliferative action of endothelium may be assessed by analysing the levels of growth factors (vascular endothelial growth factor (VEGF) and fibroblast growth factor (FGF) and an endogenous angiogenesis inhibitor, angiostatin, that blocks the growth of new blood vessels. The low-level laser irradiation may modulate endothelial function both directly but also due to the paracrine signalling following the irradiation of blood cells [4, 5]; however, the exact molecular mechanism underlying this phenomenon remain poorly understood.

LLLT has been demonstrated to increase the endothelial cell proliferation, migration as well as NO secretion. Moreover, it has been shown that activation of the PI3K/Akt pathway was a critical step for the elevated for eNOS expression upon LLLT. Furthermore, the iNOS expression and VEGF synthesis were shown to be upregulated by LLLT at the level of transcription possibly via the PI3K signalling pathway [6, 7].

Similarly, the long term effects of LLLT on profile of endothelial function, and subsequently cardiovascular risk remain unknown and most of the studies conducted so far were focused mostly on the short term outcome. Furthermore, the effect of LLLT on the paracrine interaction between streaming blood and endothelial cell layer in the vessels. Hence, the aim of the study was an attempt to evaluate the effect of the transdermal LLLT on profile endothelial function and to address the question regarding possible paracrine response to the changes in the levels of vasoactive molecules in streaming blood.

## Material and methods

### Study group

In this study *n* = 40 healthy volunteers at age of 20–40 years were enrolled, where *n* = 30 (14 female and 16 male, mean age mean age 30 ± 5 years) constituted the laser irradiated (laser group (LG)). The remaining 10 subjects (6 women and 4 men, mean age 28 ± 5 years) were the control group (CG).

The recruited subjects were young and without a significant past medical history. Exclusion criteria for the study were disorders that may affect endothelial function, such as cardiovascular disease (hypertension, diabetes mellitus, peripheral artery disease, heart failure) and cardiovascular events in the past (stroke, transient ischemic attack (TIA), myocardial infarction). Subjects with an active or chronic inflammatory process (acute or chronic infectious diseases), endocrine diseases, autoimmune diseases, haematological diseases, neoplastic disorders were also excluded from the study. Subjects with other factors potentially interfering with the obtained results (dermatological abnormalities, reduction of blood flow in the radial artery, drug therapy affecting the endothelial function) were not enrolled to the study.

### Study procedure

Each participant of the LG underwent the laser irradiation of the skin located above the course of the radial artery with 24-h intervals between procedures for three consecutive days. We have chosen the radial artery as the easily accessible and located in a relatively safe for irradiation body area.

Before starting the illumination, 40 ml of blood had been drawn from the antecubital vein from each subject for the subsequent biochemical analyses. The second blood drawing was conducted on the fourth day of the study, 24 h after the last irradiation.

Blood samples from the CG were drawn in the same time intervals.

### Laser probe and irradiation procedure

A semiconductor laser LS 808/2000 (Laser Secura, Poland) issuing the radiation of a wavelength of λ = 808 nm and a maximum power of 2 W was used in this study.

An element-emitting radiation was permanently, optically connected (“pigtailed”) with optical fibre. The end of the optical fibre was designed to work from an approximate distance of 20 cm, while the area of exposure was a circle with approximately 2 mm in diameter. During the study, the energy dose of one irradiation procedure was 20 J/ day, a total dose of 60 J with an application of radiation of 50 mW. The energy density was 1.6 W/cm^2^.

In order to validate the power and radiation energy, a PMD100D metre (Thorlabs Ltd., UK) was used prior to the study. In the course of the study, the radiation energy and the power stability were measured twice in order to avoid unreliable results. The wave length of radiation was controlled using an Optical Spectrum Analyzer AQ6370C (Yokogawa Electric Corporation, Japan).

After determining the location of the radial artery by palpation about 2 cm proximally to the styloid process of the radial bone (located ca. 3 cm proximally to the wrist line), terminal points were marked on the skin on the palpated location and 4 cm directed proximally along the forearm. Irradiation was performed by providing the energy with slow sweeping method along the course of the artery with the sweeping rate 0.5 Hz.

### Biochemical tests

During the study, the following biochemical parameters were measured:

- marker of endothelial oxidative stress—reduced glutathione (GSH)

- metabolites of the nitric oxide pathway—ADMA, SDMA (the substrate’s analogues for endothelial nitric oxide synthase, eNOS; where ADMA is an endogenous competitive inhibitor of eNOS), l-Arginine (a substrate for eNOS), cGMP (a second messenger in the intracellular signal transduction initiated by NO).

- endothelial angiogenic factors—VEGF, FGF, angiostatin.

The reduced glutathione was measured in whole blood using a colorimetric assay BIOXYTECH GSH/GSSG-412. Plasma concentrations of l-arginine, asymmetric dimethylarginine (ADMA) and symmetric dimethylarginine (SDMA) were measured by high-performance liquid chromatography (HPLC), and precolumn derivatization was measured with *o*-phthaldialdehyde (OPA) by a previously published method [8]. Plasma concentrations of cGMP, serum VEGF and FGF were measured using commercial ELISA kits (R&D Systems Europe Ltd, United Kingdom). Angiostatin in the serum was determined using ELISA kit ELH-Angiostatin (RayBio Inc., USA). Tests were conducted according to the manufacturer’s instructions and the samples were stored at −80 °C until analysis.

### Statistical analysis

Statistical analysis was performed using the Statistica 10.0 Stat Soft® software. For analysing unpaired variables, after preliminary analysis of normal distribution and homogeneity of variance, the Shapiro Wilk’s and Leven’s tests were performed. The significance of the differences in mean values were then analysed using the Mann–Whitney *U* test for non-parametric variables or *t* test for parametric ones. For paired variables, dependent on the parametricity, *t* test for matched variables or signs test and Wilcoxon’s test were conducted, as appropriate. Values of *p < 0.05* were considered as statistically significant.

## Results

No significant differences between the two groups regarding age (LG 30.03 ± 5.05 vs. CG 27.82 ± 4.65 years; *p = 0.40*), sex (women/men ratio at both groups, *p = 0.72*) nor BMI (LG 22.67 ± 2.62 vs. CG 22.44 ± 2.27 kg/m^2^; *p = 0.67*) were observed.

The mean values of analysed biochemical parameters between the two groups were similar, both at baseline and at the endopoint (Table 1). No significant changes in the analysed parameters were observed in the CG when comparing the baseline and endpoint examinations (Table 1).

**Table 1:** Comparison of the analysed biochemical parameters between groups at particular steps of the study protocol

Parameter (mean ± SD)	LG baseline *N* = 30	LG endpoint *N* = 30	LG (baseline vs. endpoint) *p* value	CG baseline *N* = 10	CG endpoint *N* = 10	CG (baseline vs. endpoint) *p* value	LG vs. CG baseline *p* value	LG vs. CG endpoint *p* value
VEGF [pg/ml]	307.34 ± 227.90	309,74 ± 241,89	0.86	285.41 ± 172.38	285.61 ± 150.20	1.00	0.85	0.76
FGF [pg/ml]	2.97 ± 2.39	2.63 ± 1.87	0.20	2.12 ± 0.95	2.77 ± 2.21	0.55	0.34	0.85
Angiostatin [pg/ml]	265.22 ± 151.43	255.16 ± 228.68	0.002	209.52 ± 37.03	321.31 ± 331.42	0.27	0.20	0.49
GSH [μmol/l]	769.41 ± 145.37	815.15 ± 151.69	0.0045	845.85 ± 173.42	708.13 ± 176.82	0.13	0.18	0.07
cGMP [pg/ml]	79.79 ± 28.18	78.40 ± 28.13	0.86	80.04 ± 27.10	73.46 ± 30.33	0.11	0.98	0.64
SDMA [μmol/l]	0.24 ± 0.05	0.24 ± 0.03	0.66	0.23 ± 0.04	0.24 ± 0.06	0.75	0.43	0.97
ADMA [μmol/l]	0.38 ± 0.05	0.37 ± 0.04	0.93	0.35 ± 0.05	0.35 ± 0.04	0.75	0.12	0.97
l-arginine [μmol/l]	35.42 ± 9.36	37.13 ± 8.60	0.13	31.17 ± 8.37	36.01 ± 11.04	0.75	0.34	0.92

LG laser group, *CG* control group, *VEGF* vascular endothelial growth factor, *FGF* fibroblast growth factor, *GSH* glutathione, *SDMA* symmetric dimethylarginine, *ADMA* asymmetric dimethylarginine

A significant increase in the reduced glutathione level in LG was noted following the LLLT (769.41 ± 145.37 vs. 815.15 ± 151.69 μmol/l respectively; *p* = 0.045).

LLLT resulted in a significant decrease in the angiostatin levels 265.22 ± 151.43 vs. 255.16 ± 228.68 pg/ml respectively; *p* = 0.002).

No significant differences between other parameters in LG were observed (Table 1).

## Discussion

In this study the effect of LLLT on endothelium and the platelet aggregation was measured using the biochemical and platelet aggregation parameters. Taken into account that the studies conducted so far were only experimental and related mostly to the cell cultures or experimental animal models, this is the first study to analyse the long-term effect of laser irradiation on the elements mentioned above in a clinical setting.

In this study, a radiation wavelength of λ = 808 nm was used since such range is located within the optical frame (about 550–1000 nm) and is relatively poorly absorbed by tissues. The greater values of the radiation wavelengths have relatively the highest tissue penetration. The total dose of radiation used in the experiment is strictly connected with the number of exposures. It is assumed that a single dose of radiation equals 20 J. Looking into the anatomical features and taking into account the possibility of the deepest tissue penetration with applied radiation wavelength, the most available artery was chosen. Based on the literature review, there are no studies assessing the most effective dose acting locally on endothelium and blood flow.

Considering the dose absorbed by the peripheral tissues, the applied energy levels in this study were much higher than those ones used in experimental studies on cell cultures or small laboratory animals.

### Reduced glutathione

Obtained results of biochemical parameters have shown that low-level laser irradiation leads to an increase in reduced glutathione, which acts as a sulphide buffer. It may be postulated that low-level laser modulation of the reduced glutathione levels could affect the generation of reactive oxygen species. There are a few reports confirming the antioxidant effect of LLLT based on the changes in the redox status [9, 10].

It appears that this effect depends on a specific dose of irradiation as well as its wavelength. In the study by Silveir et al. conducted in an animal model, the He–Ne laser (wavelength of λ = 660 nm) of energy density of 1 J/cm^2^ as well as 3 J/cm^2^ induced the reduction of oxidative stress. On the other hand, the GaAs laser with a dose of 3 J/cm^2^ caused an expected effect [11].

Parameters of laser irradiation used in this study (wavelength 808 nm, energy 20 J on exposure) have also been proven to be successful in achieving an anti-oxidative effect. In this case, the LLLT contributed to changing in the level of reduced glutathione. So far, very few studies demonstrated the relationship between LLLT and GSH in the context of antioxidant action. The de Lim et al. study carried out on rats could be an example. After the low-level laser irradiating, an increased level of reduced glutathione was observed (wavelength λ = 660 nm, power 30 mW, energy 5.4 J), which suggests antioxidant properties of the irradiation [12]. The results of this study prove the effect of LLLT on the GSH levels in human, which was previously observed only in animal models.

### Growth factors

Low-level laser radiation is presently considered to induce cell proliferation. Numerous in vitro studies conducted on various types of cell cultures, such as fibroblasts, endothelial cells, keratinocytes, myoblasts and osteoblasts [13–15] confirm the thesis. The studies report that LLLT induces proliferation of endothelial cells due to the growth factor release such as VEGF [16, 17] or FGF [18, 19]. The Goralczyk et al. study demonstrated that LLLT of HUVEC (human umbilical vein endothelial cells) induces a proliferative effect (wavelength λ = 635 nm, energy density of 2 and 4 J/cm^2^) [16].

Furthermore, the Martignango et al. study indicated that irradiation with a wavelength of λ = 904 nm and energy density of 2 and 3 J/cm^2^ used on murine fibroblasts increases the VEGF gene expression, which induces cells proliferation [20]. A similar effect was observed during irradiation of human keratinocytes with a laser of a wavelength of λ = 780 nm, and it was most noticeable by applying radiation energy equal to 1.5 J/cm^2^ [21].

The LLLT influence on changes in growth factors (VEGF, FGF) was measured in this study. There were no significant differences in their concentration after biostimulation. Insignificant increases in average levels of VEGF after LLLT were observed. Noteworthy, in the study by Derkacz et al. conducted on a clinical material, no increase in VEGF after using intravascular arteries irradiation was observed. However, intravascular irradiation caused a significant decrease of FGF levels, which was not observed in this study [17]. Similar observations relating to a lack of changes in VEGF levels may be a consequence that both studies were conducted on humans using the same wavelength (808 nm).

Differences in the response of FGF to LLLT may result from limited bioavailability of the percutaneous irradiation method when comparing to the intravascular one.

### Angiostatin

A significant reduction in the level of angiostatin in the laser group in response to LLLT was shown. Angiostatin is a well-known angiogenesis inhibitor [22, 23]. Taking into account the results, we can assume that LLLT decreases the level of angiostatin, and causes a shift of the balance favouring the pro-angiogenic activity. Therefore, LLLT may indirectly promote the angiogenesis. A few reports confirm this statement, although stimulation of angiogenesis is associated rather with an increase in iNOS activity as well as in VEGF levels. The influence of LLLT on the concentration of angiostatin was not studied so far [24, 25].

The Zaidi et al. study demonstrates that LLLT stimulates angiogenesis through the angiomotin increase and angiostatin decrease in sclerodermic mice. A 14-day exposure to laser radiation contributed to the formation of collateral circulation in the limbs, which were previously hypoxic [20]. In the available literature, there were no more data regarding the relationship between LLLT and the level of angiostatin. The results confirm the hypothesis that LLLT should not be used in human with malignancies since the angiostatin deficiency may disinhibit the uncontrolled angiogenesis [26].

### The effect of LLLT on the nitric oxide metabolism

In this study we intended to assess the effect of LLLT on the metabolism of nitric oxide. The analysis of parameters associated with the NO metabolic pathway (ADMA, SDMA and l-A rginine) were used for this purpose. However, there were no significant differences after the irradiation procedure. Nevertheless, numerous reports indicate beneficial effects of low-level laser irradiation on the bioavailability of NO [6, 7, 27, 28].

The authors analysed different parameters associated with the metabolism of nitric oxide from those ones found in this study. Our study analyses the relatively long-term effects of the high power density. In the literature, NO is shown to be released immediately and to stay for at least a few hours after irradiation [29]. Therefore, the results of this study do not oppose previous findings but look at a different time frame.

It is noted in particular, that LLLT increases the gene expression of the nitric oxide synthase. The Lohr et al. study shows that in case of hypoxia, LLLT (wavelength of λ = 670 nm) stimulates the secretion of NO [28]. Similarly, Osipov et al. showed that the photolysis of nitrosyl complexes in haemoglobin is a source of NO [30]. In the present study, we investigated the effect of LLLT on the concentration of ADMA. The presence of numerous publications highlights the importance of ADMA in the development of cardiovascular disease in relation to endothelial dysfunction. There is currently no data relating to the direct effects of LLLT on ADMA levels, which contributed to our observations. However, those studies confirm numerous beneficial effects of LLLT on the metabolism of NO that presumably takes place in a different mechanism than the one demonstrated in this study.

### LLLT and endothelial and platelet function

A vast majority of the studies on the effect of LLLT on endothelium were conducted in an in vitro model, and there are only scarce studies regarding the modulating role of LLLT on human endothelium and its proliferation [13, 21, 31, 32]. In an ex vivo study conducted on human material, it has been shown that LLLT may modulate vascular tone of the coronary arteries and left internal mammary artery following their previous pharmacologically induced contraction [33]. Some studies postulate that this effect might be mediated by changes in the nitric oxide metabolism, which is opposed by the results of our study [6, 7].

The effect of LLLT on platelet function is also a matter of several studies and controversies. In study by Brill et al. the effect of LLLT was varying dependent on the wave length and the HeNe laser with the λ = 632 nm and power of 7 mW was associated with decreased platelet aggregation induced by TRAP (the thrombine receptor agonist peptide) [34, 35]. The effect was also explained by changes in the nitric oxide metabolism, as assessed by cGMP levels. We used the wave length of λ = 808 nm, which could explain different results observed in our study.

### Study limitations

The study limitations resulted from the test procedure itself. During the study, only one wavelength of the laser radiation was used. Other radiation wavelengths may potentially have a different effect on the organism, including the endothelial function. Using the described method, it is not possible to estimate the exact dose of radiation that reaches the endothelium. However, other irradiation methods would involve an invasive procedure, which would be difficult to justify on ethical ground. Due to the small endothelial area subjected to laser irradiation, the observed effects were rather paracrine via streaming blood than exerted directly on the whole endothelium.

## Conclusions

Low-level laser radiation modulates the endothelial function by increasing its anti-oxidative and angiogenic potential. Administration of LLLT (λ = 808 nm, total dose 60 J) resulted in no significant changes in parameters of the NO metabolic pathway 24 h after LLLT irradiation.
